# Association of postoperative nausea/vomiting and pain with breastfeeding success

**DOI:** 10.1186/s13741-017-0075-2

**Published:** 2017-11-22

**Authors:** Ramon Abola, Jamie Romeiser, Suman Grewal, Sabeen Rizwan, Rishimani Adsumelli, Ellen Steinberg, Elliott Bennett-Guerrero

**Affiliations:** Stony Brook Medicine, Department of Anesthesiology, HSC-4-060, Stony Brook, NY 11794 USA

**Keywords:** Breastfeeding, Postoperative pain, Postoperative nausea and vomiting (PONV), Prelabor cesarean delivery

## Abstract

**Background:**

Successful breastfeeding is a goal set forth by the World Health Organization to improve neonatal care. Increasingly, patients express the desire to breastfeed, and clinicians should facilitate successful breastfeeding. The primary aim of this study is to determine if postoperative nausea and vomiting (PONV) or postoperative pain are associated with decreased breastfeeding success after cesarean delivery.

**Methods:**

This is a historical cohort study using the Stony Brook Elective Cesarean Delivery Database. Self-reported breastfeeding success at 4 weeks postoperative was analyzed for associations with postoperative antiemetic use and postoperative pain scores. Breastfeeding success was also analyzed for associations with patient factors and anesthetic medications.

**Results:**

Overall, 86% of patients (*n* = 81) who intended on breastfeeding reported breastfeeding success. Breastfeeding success was not associated with postoperative nausea or vomiting as measured by post anesthesia care unit antiemetic use (15% use in successful vs. 18% use in unsuccessful, *p* = 0.67) or 48-h antiemetic use (28% use in successful group vs 36% use in unsuccessful group, *p* = 0.732). Pain visual analog scale scores at 6, 12 and 24 h postoperatively were not significantly different between patients with or without breastfeeding success. Breastfeeding success was associated with having had at least 1 previous child (86% vs 36%, *p* < 0.001). Patients with asthma were less likely to have breastfeeding success (45% vs 4%, *p* = 0.002).

**Conclusions:**

Efforts to improve PONV and pain after cesarean delivery may not be effective in improving breastfeeding success. To possibly improve breastfeeding rates, resources should be directed toward patients with no previous children and patients with asthma.

## Background

Successful breastfeeding is a goal set forth by the World Health Organization to improve neonatal care (WHO, 2011). The American Academy of Pediatrics (AAP) recommends that children are exclusively breastfed for the first six months of life (So, 2012). Increasingly, patients express the desire to breastfeed, and clinicians should facilitate successful breastfeeding. Previous studies have reported that cesarean deliveries were associated with a lower breastfeeding rate compared to vaginal deliveries (Prior et al., 2012). This is important because cesarean delivery rates have steadily increased over the past three decades in the United States. Most recently, the Centers for Disease Control reported a cesarean delivery rate of 32.2% in the United States (Osterman & Martin, 2014).

After cesarean delivery, routine postoperative care can delay maternal bonding with the infant. A delay in initial breastfeeding has been associated with less successful breastfeeding (Lin et al., 2011; Wallwiener et al., 2016). Our primary objective was to determine if postoperative nausea and vomiting (PONV) after cesarean delivery is negatively associated with breastfeeding success. We also evaluated if postoperative pain affects breastfeeding success. We hypothesized that patients who had more PONV and pain would be less successful at breastfeeding.

## Methods

After institutional and ethics approval, a registry was created for patients who presented for prelabor (elective or scheduled) cesarean delivery. Information collected included patient demographics and comorbidities, indications for cesarean delivery, intraoperative management and postoperative outcomes including length of stay, pain scores and use of antiemetics. Patients were contacted by telephone 4 weeks after delivery in order to complete a brief telephone survey. Two questions related to breastfeeding were asked: 1. Did you intend on breastfeeding? 2. Were you successful with breastfeeding? Successful breastfeeding was subjectively defined by each individual patient.

At our institution, patients who present for elective/scheduled cesarean delivery typically receive a spinal anesthetic with bupivacaine (12 mg), fentanyl (10 mcg) and morphine (0.2 mg). A phenylephrine infusion and a fluid bolus of lactated ringer’s are used to decrease the incidence of spinal hypotension. Antiemetics and supplemental intravenous analgesics or sedatives are given as needed. After delivery, patients receive an infusion of oxytocin (20 units over 1 h and then 5 units/h for 4 h). In our post anesthesia care unit (PACU), patients can receive ketorolac (30 mg) for postoperative pain. The postoperative analgesia regimen includes intrathecal morphine from the spinal anesthetic, oral acetaminophen, oral ibuprophen and oxycodone as needed.

The data were collected prospectively by anesthesia providers and entered into a Microsoft Access database. This project was authorized by the Stony Brook Medicine Division of Medical and Regulatory Affairs Office as part of the Surgical Quality Improvement Program (SQIP). Following de-identification and extraction of all SQIP patient records, a data analysis was performed as part of the Surgical Quality Data Users Group (SQDUG). The SQIP/SQDUG protocols were approved by our institution’s investigational review board (Committees on Research Involving Human Subjects [CORIHS] #170753-9 – Stony Brook University).

Fisher’s exact tests and Wilcox rank sum tests were used to examine association between breastfeeding success and the following variables: comorbidities, ASA classification, intraoperative sedative and analgesic medications, PACU antiemetics, 48 h antiemetics, parity, and Visual analog scale (VAS) score at 6, 12 and 24 h after surgery. All calculations were performed at an alpha of 0.05 using SAS 9.4 Software **©** (SAS Institute Inc., Cary, NC).

## Results

Information was collected for 391 patients who presented to Stony Brook University Medical Center between October 2013 and September 2014 for elective or scheduled cesarean delivery. Overall, 132 patients completed a post-discharge telephone survey 4 weeks after surgery (34% survey completion). Ninety-four surveyed patients (71%) stated that they intended to breastfeed. Further analysis was performed on this cohort who intended to breastfeed. Eighty one patients (86%) reported successful breastfeeding and 11 patients (11%) reported they were unsuccessful with breastfeeding; two did not report on their success (2%).

With regard to our primary objective, breastfeeding success was not associated with PONV. Breastfeeding success was not associated with PACU antiemetic use (15% use in successful vs. 18% use in unsuccessful, *p* = 0.67). Breastfeeding success was not found to be associated with 48-h antiemetic use (28% use in successful vs. 36% use in unsuccessful, *p* = 0.73, Table 1). Pain visual analog scale scores (VAS) at 6, 12 and 24 h postop were not significantly different between patients with or without breastfeeding success (Table 2).

**Table 1:** Breastfeeding success and antiemetic use, demographics, comorbidities, and delivery characteristics

Patient Characteristics	Unsuccessful Breastfeeding	Successful Breastfeeding	*p* value
Total Number of Patients	*n* = 11	*n* = 81	
Antiemetic Use			
PACU Antiemetics	2 (18.2%)	12 (14.8%)	0.67
48 Hour Antiemetics	4 (36.4%)	23 (28.4%)	0.73
Demographics			
Age (Years) - Median (IQR)	33 (28 - 34)	33 (28 - 36)	0.66
Height (cm) - Median (IQR)	160 (157 - 165)	162 (157 - 167)	0.44
Weight (kg) - Median (IQR)	93 (70- 100)	84.5 (74 - 94.5)	0.68
BMI (kg/m^2^) - Median (IQR)	35.4 (27.1, 39.6)	32 (28.3 - 36)	0.53
Ethnicity (Caucasian vs. Non)	7 (63.6%)	53 (65.4%)	1
Parity (≥1)	4 (36.4%)	70 (86.4%)	<0.001
ASA Classification			
- ASA I	2 (18.2%)	32 (39.5%)	0.32
- ASA II	8 (72.7%)	45 (55.6%)	
- ASA III	1 (9.1%)	4 (4.9%)	
Gestational Age (Weeks) - Median (IQR)	39 (38 - 39)	39 (39 - 39.2)	0.07
Comorbidities			
Any Co-Morbidity	10 (90.9%)	41 (50.6%)	0.02
History of PONV	0 (0%)	13 (16.5%)	0.35
Diabetes	3 (27.3%)	6 (7.4%)	0.07
Asthma	5 (45.5%)	5 (6.2%)	0.002
Other Condition	4 (36.4%)	31 (38.3%)	1
Delivery Characteristics			
*Indications for cesarean delivery*			
Repeat Cesarean	4 (36.4%)	60 (75.0%)	0.01
Malpresentation	1 (9.1%)	10 (12.7%)	1
Macrosomia	2 (18.2%)	2 (2.5%)	0.07
Multiple gestation	1 (9.1%)	4 (5.0%)	0.48
Anesthetic technique			
- Spinal	8 (72.7%)	80 (98.8%)	0.005
- Combined Spinal Epidural	3 (27.3%)	1 (1.2%)	
Any Intraoperative Sedatives or Analgesics	2 (18.2%)	19 (23.5%)	1
Any Intraoperative Antiemetics	6 (54.6%)	50 (63.3%)	0.74

**Table 2:** Breastfeeding success and visual analog scale (VAS) pain scores

	Unsuccessful Breastfeeding	Successful Breastfeeding	*P*-value, Wilcox rank sum test
VAS 6 Hours	*n* = 10	*n* = 73	
Median (Q1/Q3)	1.5 (0/4)	3 (0/4)	0.56
Mean (Upper/Lower 95%)	2.0 (0.7-3.4)	2.73 (2.1-3.3)	
VAS 12 Hours	*n* = 9	*n* = 75	
Median (Q1/Q3)	1 (0/1)	2 (0/5)	0.39
Mean (Upper/Lower 95%)	1.6 (0-3.3)	2.6 (1.9-3.2)	
VAS 24 h	*n* = 10	*n* = 80	
Mean (Upper/Lower 95%)	4.5 (2/6)	3 (1/5)	0.31
Median (Q1/Q3)	4.1 (2.2-6.1)	3.2 (2.6-3.8)	

Breastfeeding success was positively associated with having previous children: 86% of those who reported success had at least one previous child; just 36% of those who were unsuccessful had at least one previous child (*p* < 0.001). Patient comorbidities had a negative affect: 91% of those who were not successful had some type of co-morbidity. This proportion was significantly lower (51%) in those who reported success (*p* = 0.02). Specifically, a higher proportion of those who reported unsuccessful breastfeeding were asthmatic (46% vs. 6%, *p* = 0.002). Diabetes also trended toward significance in this direction. Breastfeeding success was not found to be associated with ASA score, or need for intraoperative sedative or analgesic medication (*p* = 1). However, there was a difference in anesthetic technique; a higher proportion of those who reported unsuccessful breast feeding had a combined spinal/epidural compared to those who reported successful breast feeding (27% vs. 1%, *p* = 0.005).

Surveyed patients were compared to non-surveyed patients (*n* = 259) for differences in demographics, pre and post -operative information. Aside from having a lower ASA score in the surveyed group, there were no discernible differences (not shown).

## Discussion

Our analysis found no association between breastfeeding success and PONV or postoperative pain scores. We hypothesized that a better recovery profile would facilitate success at breastfeeding and that PONV and pain might make women more likely to give up trying to breastfeed. A previously published survey study demonstrated that mothers with increased pain after cesarean delivery had increased problems with breastfeeding (Karlstrom et al., 2007). We could find no data in the medical literature that correlated breastfeeding success with maternal PONV.

Our study found that mothers with previous children were more likely to be successful at breastfeeding, which is consistent with prior studies (Sutherland et al., 2012). It is interesting to note that mothers with asthma were much more likely to be unsuccessful at breastfeeding. It is unclear from our study if asthma causes a physical limitation that affects breastfeeding, or if asthma is a surrogate for demographic differences such as tobacco abuse or lower socioeconomic status. Lower socioeconomic status has been associated with lower breastfeeding rates (Brand et al., 2011). Future research should evaluate if improving breastfeeding rates in asthmatic mothers results in improved long-term outcomes for their children. A meta-analysis found that breastfeeding was associated with decreased incidence of asthma in children (Dogaru et al., 2014). Targeting breastfeeding resources toward first time mothers and asthmatic mothers may increase rates of breastfeeding success.

There are a limited number of studies that have looked at the effects of neuraxial anesthesia during childbirth on breastfeeding. Previous studies have suggested that epidural analgesia in labor is associated with lower breastfeeding rates (Baumgarner et al., 2003; Torvaldsen et al., 2006). However, a study by Halpern drew opposite conclusions demonstrating high breastfeeding rates in both patients who received or did receive labor epidural analgesia (Halpern et al., 1999). A systematic review found inconclusive evidence within the medical literature to determine the effect of epidural analgesia on breastfeeding (French et al., 2016).

A systematic review of breastfeeding worldwide reported a lower rate of breastfeeding in patients who had a cesarean versus a vaginal delivery (pooled odds ratio: 0.57 l 95% CI 0.50, 0.64, P, 0.00001). The rates of breastfeeding were also lower in patients who had a prelabor (elective/scheduled) cesarean delivery versus an emergency, in-labor cesarean delivery (prelabor odds ratio: 0.83, 95% CI 0.80, 0.86, P, 0.00001, in labor odds ratio: 1.00, 95% CI 0.97, 1.04; *P* = 0.86) (Prior et al., 2012). The authors of this review speculated on several possible reasons for these findings. The first hours after delivery are important to establish breastfeeding between a mother and neonate. Postoperative evaluations and interventions may impede the creation of this bond between mother and child. Another idea is that there may be hormones released in labor that promote breastfeeding that are not released in prelabor cesarean delivery patients. However, these authors also observed that there were no differences at 6 months between vaginal delivery and cesarean delivery in the patients who had successful early breastfeeding (Thompson et al., 2010). Patients with greater blood loss due to postpartum hemorrhage were less likely to initiate and sustain full breastfeeding. There is speculation that less successful breastfeeding in these patients may be related to delays in initial contact/bonding between mother and child.

The World Health Organization reports that the exclusive breastfeeding rate at 6 months of life for newborns worldwide is 40%. (Global breastfeeding scorecard, 2017) In the United States, two thirds of mothers breastfed in the 1900s. Breastfeeding rates declined over the twentieth century reaching a nadir in 1972 with breastfeeding rates of 22%. Factors that are associated with the declining rates of breastfeeding include the increased use of breast milk substitutes, the need to return to work away from their babies, and lack of family support (Global breastfeeding scorecard, 2017; Raffle et al., 2011; Wolf, 2003). Breastfeeding rates have increased over the last few decades (Wolf, 2003).

Our high level of breastfeeding success may have little to do with the anesthetic management and may be more reflective of institutional policies that encourage breastfeeding. Our institution has worked toward earning a “Baby-Friendly” designation by instituting the AAP’s *Ten Steps to Successful Breastfeeding* (So, 2012). Examples of these practices at our hospital include breastfeeding in the PACU after cesarean delivery, mothers and infants “room in” remaining together 24 h a day, pacifiers are not provided to the infants, postpartum lactation consultant services are available, and breastfeeding training is completed by all nursing staff. 32% of mothers at our institution exclusively breastfed during their hospital admission in 2012. Three years later, the exclusive breastfeeding rate increased to 56% of mothers in 2015. Implementation of these breastfeeding policies has allowed for consistently high rates of breastfeeding over time at other institutions (Philipp et al., 2003).

There are several limitations of this study. This is a historical cohort study and is thus subject to bias. Our breastfeeding analysis was performed in only 94 patients with completed survey data. Inclusion of more patients may have influenced our findings. Inclusion of more patients may have influenced our findings. We performed a sample size calculation that asked how many patients we would have needed in our study to obtain statistical significance with regard to breastfeeding, antiemetic use and pain scores. We calculated that we would have needed 3000 patients to detect a statistically significant difference in antiemetic use and breastfeeding success, and approximately 500-800 patients to detect a significant difference in pain score and breastfeeding success. We cannot rule out there being some association, however, these large required sample sizes suggest that any effect is probably small, given the large number of patients likely needed.

We used antiemetics as a well-established surrogate measure of PONV, however, it is possible that some patients may have had nausea but did not receive antiemetics (Myles & Wengritzky, 2012). Our definition of breastfeeding was subjective for each individual patient. We chose a subjective definition of breastfeeding success, as there is variability to what constitutes success between patients. Breastfeeding success could be defined as 1. successful breastfeed with formula supplementation (complementary feeding), 2. breastfeeding for 6 weeks until return to work, or 3. exclusive breastfeeding for 6 months. More objective measures (breastfeeding initiation, exclusive breastfeeding, breastfeeding at 6 months) may have provided more clinically meaningful information. Further investigation into the reasons as to why 29% of our patients did not intend to breastfeed may provide better insight into how to improve exclusive breastfeeding rates.

## Conclusions

Breastfeeding success was not found to be associated with postoperative nausea and vomiting or postoperative pain. Efforts to improve PONV and pain after cesarean delivery may not be effective in increasing rates of breastfeeding success. To possibly improve breastfeeding rates, resources should be directed toward patients with no previous children and patients with asthma.

## Abbreviations

AAP: American Academy of Pediatrics
PACU: Post anesthesia care unit
PONV: Postoperative nausea and vomiting
SQDUG: Surgical quality data use group
SQIP: Surgical quality improvement program
VAS: Visual Analog Scale
